# Voluntary optimisation of antimicrobial consumption in swine and poultry production in Thailand: a policy analysis

**DOI:** 10.3389/fvets.2024.1375127

**Published:** 2024-07-10

**Authors:** Angkana Lekagul, Supapat Kirivan, Wanwisa Kaewkhankhaeng, Saowapa Khotchalai, Rodolphe Mader, Viroj Tangcharoensathien

**Affiliations:** ^1^International Health Policy Program, Ministry of Public Health, Nonthaburi, Thailand; ^2^Emergency Centre for Transboundary Animal Diseases (ECTAD), Regional Office for Asia and the Pacific, Food and Agriculture Organization of the United Nations (FAO), Bangkok, Thailand

**Keywords:** antimicrobial resistance, poultry, pig, antimicrobial, antibiotic, policy, policy analysis

## Abstract

Antimicrobial resistance (AMR) is a global health concern with significant implications on economies and health security, affecting humans, animals, food, and the environment. To tackle this issue, promoting responsible antimicrobial use in livestock production has emerged as a crucial intervention. In 2018, Thailand introduced the Voluntary Optimization of Antimicrobial Consumption (VOAC) programme, with the objective to encourage responsible antimicrobial use practises. This study aimed to analyse the context, content, process and actors of the VOAC programme. A qualitative method including document reviews and key informant interviews were applied. In-depth interviews were conducted with 18 key informants who are key stakeholders from public and private sectors involved in the policy formulation, design of policy contents and implementation of VOAC: policy makers or officers responsible for animal health (*n* = 12), animal producers (*n* = 2), animal product traders or retailers (*n* = 2), and farm veterinarians (*n* = 2). Interview transcripts were validated by informants for accuracy, and triangulated with document review findings. Deductive approach was applied for data analysis and interpretation based on Walt and Gilson’s policy analysis framework. The VOAC farm certification comprises of Raised Without Antibiotics (RWA) and Reducing Antibiotic Use (RAU), both aiming to combat AMR in food animals. Global and national factors, including increased public awareness, policy commitments, export requirements from the European Union, and international organisation advocacies, influenced the development of the programme led by the Department of Livestock Development (DLD), under the Ministry of Agriculture and Cooperatives. Collaboration with the private sector facilitated policy clarity, with implementation primarily executed through regional, provincial, and district livestock officers. Integration of the programme with the pre-existing Good Agriculture Practise certification system enabled cost-effective implementation without additional resources. In 2022, DLD official data reported 214 RWA farms (112 pig and 102 broiler), and 230 RAU farms (83 pig and 147 broiler). Incentives for farms to participate in the programme include improving corporate image and demonstrating corporate responsibility addressing AMR in food products. Recommendations include optimising certification strategies, increasing consumer awareness of RWA and RAU products and strengthening monitoring and evaluation systems.

## Introduction

Antimicrobial resistance (AMR) is one of the most critical health concerns, threatening economies and health security worldwide (1). AMR is a One Health issue as it can spread across humans, animals, food, and the environment. In particular, resistant bacteria can be transmitted from animals to humans through the consumption of animal products contaminated with antimicrobial resistant bacteria, by direct contact or indirectly through the environment (2).

One of the main selective pressures of antimicrobial resistant bacteria in food producing animals is antimicrobial use (AMU), a common practise to treat, control and prevent diseases (3). Hence, it is essential to reduce AMU or improve prudent use in animal food production globally, as called upon in the Food and Agriculture Organisation (FAO) Action Plan on AMR (4) and World Organisation for Animal Health (WOAH) Strategy on AMR and the Prudent Use of Antimicrobials (5), which are aligned with the Global Action Plan on Antimicrobial Resistance (GAP-AMR) (6). A systematic review and meta-analysis showed that interventions which restrict antibiotic use in food-producing animals are associated with a reduction in the presence of antibiotic-resistant bacteria in these animals (7). On top of regulations of antimicrobial distribution and use, many activities have been proposed to optimise AMU in animal health. For example, the European Medicine Agency (EMA) and the European Food Safety Authority (EFSA) suggested measures along the three principles ‘reduce, replace and rethink’. These measures include setting national goals for reducing antimicrobial consumption, eliminating unnecessary use by replacing them with alternative measures such as vaccines, reserving critically important antimicrobials for humans as the last choice, and rethinking livestock production systems through implementation of farming practises such as proper sanitation and biosecurity to prevent infection (8, 9).

In Thailand, AMR has been a top priority, demonstrated by the Cabinet endorsement of the National Strategic Plan on Antimicrobial Resistance (NSP-AMR) 2017–2021 in 2016. The NSP-AMR aimed to achieve a 30% reduction in antimicrobial consumption by 2021. Goal 3 of the NSP-AMR focused on promoting appropriate AMU with various interventions including prohibition of AMU for growth promotion in food animals, as well as surveillance and monitoring of AMR and AMU in livestock (10).

The swine and poultry sectors are vital components of Thailand’s livestock industry (11). In 2022, the country produced 10.8 million pigs and 300.4 million broilers, which reflect the significant scale of livestock production (12). The livestock industry in Thailand accounted for 21.1% of the total value of agricultural exports in 2020 (12). The exported-oriented industries of broilers and swine products have to adhere to requirements by imported countries. For example, the Europen Union issues certain conditions, such as low levels of farm antibiotic use, below 30 mg per kg of population correction unit (PCU) by animal species, and most antibiotic use should be for individual treatments, and restrictions on highest-priority critically important antibiotics (13).

Goal 3 of the NSP-AMR has been achieved with 36% reduction, from 658.7 mg/PCU_Thailand_ in 2017 to 421.5 mg/PCU_Thailand_ in 2020 (14). One of the initiatives of Thailand to reduce AMU has been the development and implementation of Voluntary Optimization of Antimicrobial Consumption (VOAC) certification programme, mentioned in Strategy 4 of the NSP-AMR: Prevention and control of AMR and the optimal use of antibiotics in agriculture and animals. The DLD under the Ministry of Agriculture and Cooperatives took the lead in launching the VOAC programme in 2018, which includes two farm certifications: RWA and RAU. Under the RWA certification, the use of antibiotics from birth to harvest is strictly prohibited, while the RAU certifies farm that demonstrate a reduction in antibiotic use.

The aims of this study were to analyse the context, content, process and actors of the VOAC certification programme.

## Materials and methods

### Study context

Thailand, classified as an upper-middle-income country, features an agricultural sector that contributes approximately 8% to its GDP (15). In 2022, the country produced 10.8 million pigs by 149,575 farmers and 300.4 million broilers by 31,117 farmers, which reflect the significant scale of livestock production (12). Though there is no accurate and up-to-date records on number of poultry and swine farms by their annual production capacities, a few largest scale high technology conglomerate producers are the market leaders which occupied major market especially for exports due to their technologies and capital. However, the production capacity of small farm holders are limited and mostly for local markets.

To enhance farm management practises, the Thai Department of Livestock Development (DLD) awards Good Agriculture Practises (GAP) certificates to farms that adhere to stringent standards of animal husbandry. These certified farms are mandated to engage designated veterinarians to oversee disease control, prevention, and treatment, including the judicious use of antibiotics. While GAP certification remains voluntary, it serves as a testament to the commitment towards sustainable agricultural practises. Notably, in 2019, Thailand recorded a total antimicrobial consumption of 336.3 mg/PCU in food-producing animals (16). The National Strategic Plan on Antimicrobial Resistance (NSP-AMR) has been implemented from 2016 to 2021 reflects a proactive stance, exemplified by the complete prohibition of antimicrobial use as growth promoters since 2015. In 2018, most veterinary antimicrobials in Thailand were categorised as dangerous drugs, exempt from prescription requirements but necessitating dispensation by licenced pharmacists or veterinarians at authorised pharmacies (17).

### Study design

This study was carried out in Thailand from September 2021 to July 2022 by employing a qualitative approach. Reviews of relevant policies and regulations were followed by in-depth interviews with different level of stakeholders who involved in the programmes. Triangulation of interview findings was carried out by verifying with findings from document reviews. Qualitative research methods were utilised to explore and understand the policy processes of introducing and implementing the VOAC in the swine and poultry production. Such methods allowed researchers to delve into the perspectives, experiences, and behaviours of different stakeholders (including policy makers, implementers, and the industry) involved in antimicrobial use. Furthermore, employing a case study approach (18) enabled researchers to unpack and understand the complex issue in real life setting using the case of antimicrobial consumption in swine and poultry production in Thailand.

**Figure 1:**
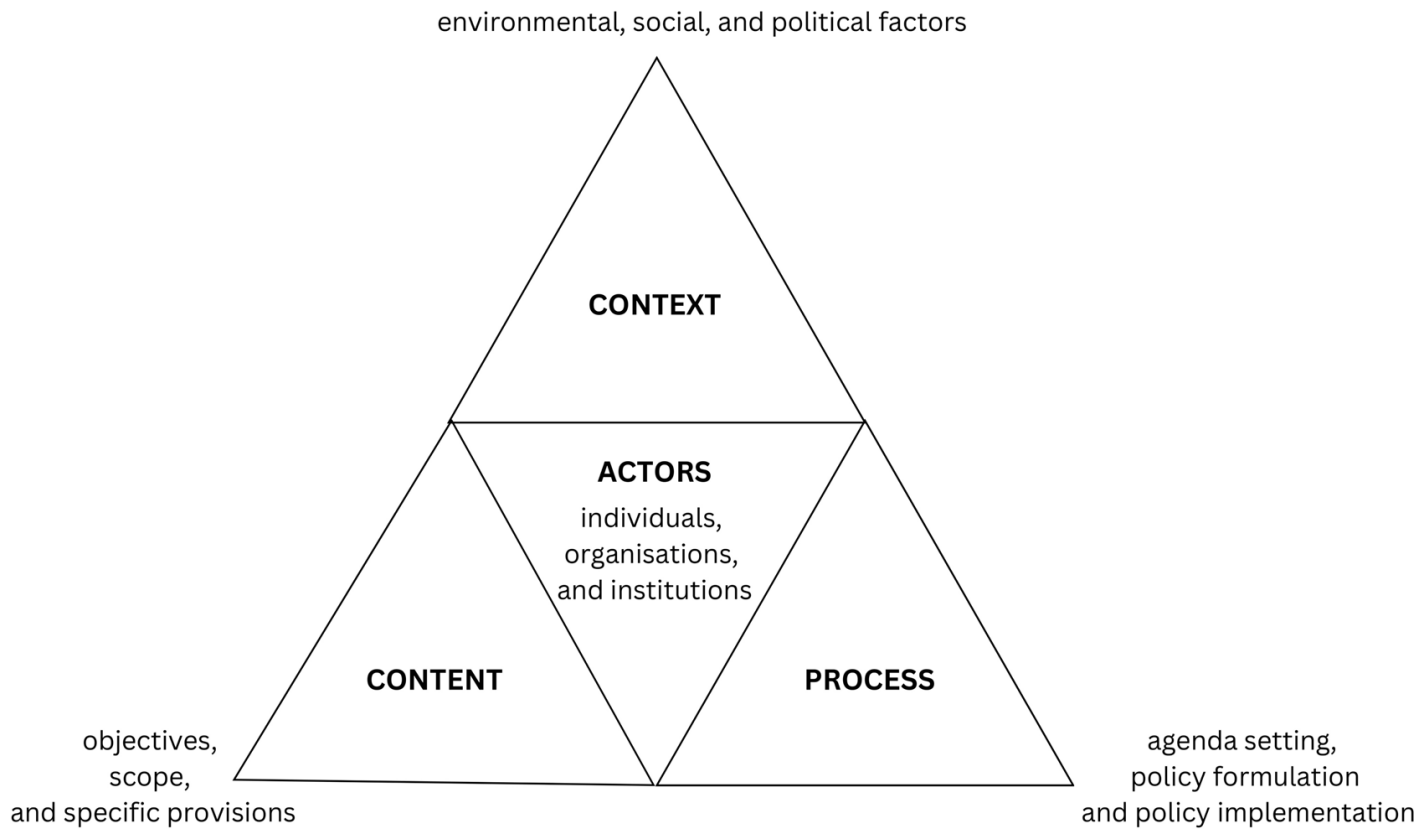
Policy analysis framework. Adapted from Walt and Gilson (19); with permission from Oxford University Press. Reforming the health sector in developing countries: the central role of policy analysis.

The policy analysis of the VOAC programme was carried out according to the framework developed by Walt and Gilson (19). This framework consists of four components: policy context, content, process and actors (Figure 1). The context component focused on understanding the environmental, social, and political factors that led to the adoption of the VOAC programme. This encompassed examining factors such as economic conditions, public opinion, scientific evidence, cultural considerations, and legal frameworks. The content of the policy was examined to gain insights into its objectives, scope, and specific provisions. The policy process was analysed in terms of agenda setting, policy formulation and policy implementation. Key actors involved in the policy process include individuals, organisations, and institutions that played various roles in policy development, decision-making, and implementation.

### Data collection

To collect the necessary information related to policy analysis, a document review was carried out, followed by key informants interviews.

#### Document review

A thorough analysis of policy documents and related regulations was conducted. The review aimed to identify and examine policies concerning the VOAC in swine and poultry production settings. Key documents included the NSP-AMR covering the period from 2016 to 2021. Additionally, documents related to RWA and RAU certifications were retrieved and reviewed to provide a thorough understanding of the regulatory framework which governs antimicrobial use in the food animal production, as well as certification reports, programme reports, and other relevant materials.

#### Key informant interviews

The key informant interviews were conducted to obtain further information about the VOAC and its RWA and RAU certifications, especially regarding their implementation. A semi-structured interview guide was designed based on the policy analysis framework by Walt and Gilson (Supplementary material 1). The interviews were conducted in Thai through video conference and audio-recorded.

On average, each interview session lasted approximately one and a half hours. The interviews took place in a conducive environment mostly in the office of participants which ensured privacy and comfort for both the interviewers and respondents. In terms of ethical consideration, while written consent is considered best practise, logistical constraints during the interviews necessitated the use of verbal consent in this study. Participants were fully informed of their rights, including the right to withdraw from the study at any point and the right to request anonymity. Participants are provided with researchers’ contact detials. Participants’ contact information was requested and provided to support follow-up and clarification of the study’s findings if needed.

A purposive sampling technique was utilised to select key informants from governmental organisations responsible for AMR policy at national and local levels including the Animal Feed and Veterinary Products Control (AFVC), Bureau of Quality Control and Livestock Product and Provincial Livestock Offices (PLO) from four purposive selected provinces. In addition, to diversify perspectives from stakeholders outside government sector, relevant private sector associations such as Animal Health Products Association (AHPA) and the Thai Feed Mill Association (TFMA), were requested to propose lists of their officers or members who could provide information about RWA and RAU certifications including animal product retailers and farm veterinarians. Furthermore, AFVC was consulted to propose names of livestock producers who participated the certifications. It should be noted that, in this study, we did not involve key informants from farms which are not in the VOAC programme.

In total, 18 key informants from the public and private sectors involved in the development and implementation of the VOAC programme were interviewed after verbal consent. They were categorised into four groups according to their respective roles (Table 1). Though we received the name and contact of eight farmers for both pig and poultry productions, only two of them agreed to be interviewed after contact and explanation about the study.

**Table 1:** Four groups of respondents’ profiles.

	Total (*N* = 18)	Gender		Age (average, range)
		Male (12)	Female (5)	
Group A: Policy makers or officers responsible for animal health	12	7	5	47 (34–61)
Group B: Animal producers or farmers	2	2	0	63 (55–71)
Group C: Animal product traders or retailers	2	2	0	55.5 (55–56)
Group D: Veterinarians (farm veterinarians)	2	2	0	59 (51–67)

### Data processing and analysis

Following the interviews, each key informant was requested to validate his/her transcription to ensure the accuracy of the information provided. This was followed by a triangulation to cross-verify findings between interviews and document reviews. Subsequently, two teams of researchers, each comprises of two researchers (AL and SK1; SK2 and WK), independently analysed the collected data, after which they discussed the findings and reach a consensus. This ensures accuracy and consistency of interpretation between the two teams. A coding framework and pre-established codes developed by researchers provided a structured approach to categorise and analyse the data from document reviews and insights from key informant interview transcripts. The framework was structured in order to facilitate a deductive approach along the line of the policy process developed by Walt and Gilson. The policy process comprised of policy context, policy processes and policy contents. See details in Supplementary material 1.

## Results

Our findings were organised under the policy process framework. Deductive approach was based on three major themes (a) Policy context with three subthemes: (i) regulatory framework, (ii) international regulatory compliance for accessing export markets, and (iii) NSF-initiated RWA certification; (b) Policy process including (b.1) agenda setting with two subthemes: (i) news and media attention: public awareness of AMR, (ii) global and national policies on the optimal use of antibiotics in animal production; (b.2) policy formulation with two subthemes: (i) government, private and academia collaboration, (ii) integration into NSP-AMR; (b.3) policy implementation with five subthemes: (i) role of the veterinary authority, (ii) agro-industrial participation, (iii) certification process and implementation, (iv) GAP certification integration and monitoring mechanisms, (v) implementation challenges; and (c) Policy content with two subthemes: (i) alignment with NSP-AMR goal, (ii) certification objectives and criteria. Stakeholder involved in the policy processes were discussed under relevant themes and subthemes. Table 2 summarised of theme and subthemes generatefrom this study.

**Table 2:** Summary of theme based on policy analysis framework.

Themes based on policy analysis theme	Sub-themes
a. Policy context	Regulatory Framework International regulatory compliance for accessing export markets NSF-Initiated RWA certification
b. Policy process:	
b.1. Agenda setting	News and media attention: public awareness of AMR Global and national policies on the optimal use of antibiotics in animal production
b.2. Policy formulation	Government-Private Sector-Academia collaboration Integration into NSP-AMR
b.3. Policy implementation	Role of the veterinary authority Agro-industrial participation Certification Process and Implementation GAP certification integration and monitoring mechanisms Implementation challenges
c. Policy content	Alignment with NSP-AMR goal Certification objectives and criteria

### Policy context

Thailand is a major livestock producer. It is also a major food exporter, ranked 15th globally and contributing to 2.3% of the total world food market (20). Since 2002, all Prime Ministers have declared that Thailand aims to be ‘the Kitchen of the World’, which reflects strong political commitment to exporting quality agricultural products (21).

In recent years, Thailand has enacted a number of regulations to support more prudent AMU in the animal sector. Since 2015, the use of all antimicrobials for growth promotion has been banned. Historically, the Thai Food and Drug Administration (FDA) classified most veterinary antimicrobials as ‘dangerous drugs’, which did not legally require a prescription though need to be dispensed by a licenced veterinarian or a pharmacist. Between 2019 and 2020, the FDA reclassified certain groups of antimicrobials, including cephalosporins, polymyxins (e.g., colistin), quinolones, and macrolides, as ‘specially-controlled medicines’. This reclassification mandates that a prescription from a veterinarian is required for their dispension and use (22). Furthermore, DLD, under mandate of the Animal Feed Quality Control Act 2015, issued a notification in 2019 which mandates that dispensing and using medicated feed (i.e., feed mixed with medicines) requires a prescription (23). In addition, to address public health concerns, DLD also prohibited colistin and fluoroquinolones from mixing in animal feed in 2019, both of which are classified as highest priority critically important antimicrobials by the World Health Organisation (WHO) (24, 25).

In Thailand, the livestock production sector is predominantly controlled by large companies which apply vertical integration throughout the production-consumer chains (26). These companies are major producers of poultry and swine and own slaughterhouses and retail outlets for selling their products. Thailand is the world’s largest exporter of frozen chicken, commanding a substantial 28.9% market share in terms of volume (27). Importing countries have established food safety regulations on meat products that exporters need to follow.

For example, the European Union set up the standard for food importer requirement; consumers also shape food quality and safety as is the case of EU Regulation 2018/848 (28). Paragraph 43 and its Annex II paragraph 1.5.1.3 and 1.5.1.4 which enforces export of organic products and labelling as organic products, shall not use antibiotics for preventive treatment and growth promotion.

RWA certification is an initiative by the National Sanitation Foundation (NSF) (29). The NSF develops standards for public health and certification programmes that help protect the quality of food, water, consumer products and environment. The DLD’s RWA certification programme was introduced while the NSF was already providing the NSF RWA certification for Thai farms. Informants from RWA-certified producers informed us that although the DLD’s RWA certification is not recognised by the EU, they still participate in the programme because they are already implementing RWA through the NSF.

### Policy process

#### Agenda setting

Various factors shaped the VOAC agenda: public awareness of AMR, global and national policies on the optimal use of antibiotics in animal production, and demand from international consumers.

##### News and media attention: public awareness of AMR

In 2016, there was widespread mass media attention on the presence of colistin resistant bacteria (carrying plasmid mediated *mcr-1* gene) in pigs in three provinces of Thailand, and the use of colistin in pig farms and human health consequences. Public concerns were raised about the possibility of AMR transmission from pigs to humans, leading to panic in the public about AMR in the food chain. The use of colistin, one of the reserved antibiotics, in pig farms was pointed out as the main driver for the presence of colistin resistant bacteria in pigs. Subsequently, the Minister of Public Health, the Secretary General of FDA and the Director General of DLD issued a press release confirming that FDA and DLD shall reclassify colistin for veterinary use from ‘dangerous drugs’ to ‘specially-controlled medicines’, which was finally accomplished in 2019.

##### Global and national policies on the optimal use of antibiotics in animal production

Several international policy documents, such as the GAP-AMR (8), the political declaration on AMR made at the United Nations General Assembly High-Level Meeting in 2016, the FAO Action Plan on AMR (4) and the WOAH Strategy on AMR and the Prudent Use of Antimicrobials (5), and the NSP-AMR recommend the optimal use of antibiotics in animal production. The NSP-AMR followed the ‘One Health’ approach which links various sectors and actors in defence of human, animal and environmental health (32).

All of the key informants from the private sector said their industry were aware of the GAP-AMR. A key informant from a private food-producing company informed that they have signed a commitment to support the United Nations efforts to combat AMR at the One Health Summit in 2016 (33).

*‘…In 2016, our company attended the One Health meeting and adopted five policies to jointly solve the problem of AMR such as responsible use of antibiotics under veterinary supervision, non-use of antibiotics for growth promotion, promoting raised animals without antibiotic and monitoring AMR at farms annually, in compliance with DLD. The company signed a commitment to support the United Nations efforts to combat AMR on October 16, 2017…’* [C01, male, age 55 years].

#### Policy formulation

The AFVC, at central level, is the key implementing agency for Strategy 4 of the NSP-AMR (AMR prevention and control and antimicrobial stewardship in agriculture and animals). Within this strategy, there is a sub-committee where members were representatives from the government, the private sector and academia. At a sub-committee meeting in 2017, the AFVC discussed the possibility of initiating the RWA and RAU certifications in Thailand.

In 2018, the RWA and RAU certifications were included in the operational plan of the NSP-AMR. On 30 April 2018, DLD signed the Memorandum of Understanding with livestock producers and food retailers for collaboration on RWA and RAU certifications on a voluntary basis. The AFVC set up the farm criteria in each certification, see Table 3.

**Table 3:** Comparison of objectives and requirements of the RWA and RAU certifications.

	RWA	RAU
Objectives	To control, prevent, reduce or slow down the problem of AMR in animals To create animal producers’ awareness on antibiotic-free animal production To build consumer’s confidence in food safety in antibiotic-free livestock products To provide consumers choices of antibiotic-free livestock products	To control, prevent, reduce or slow down the problem of AMR in animals To create animal producers’ awareness on antibiotic reduction in animal production To build consumer’s choices of livestock products with rational use of antibiotics
Criteria	Health supervision by farm veterinarian (Farms with GAP certification^*^ for pig fattening farm, it must receive β-Agonist free certification from DLD.) No antibiotic use in any form at any stage of animal production Water for animals without antibiotic contamination Sick animals are treated by antibiotic and isolated outside the system/ farm. Anticoccidials, vaccine or alternatives to antibiotics which are registered can be used. Documents for traceability are kept and ready for inspection. Samples including water, feed and products must be tested annually for antibiotic contamination.	Health supervision by farm veterinarian Volume of antibiotic use must be reduced (compare between previous and current production cycle) Documents for traceability are kept and ready for inspection

^*^The GAP certificate for pig farms was introduced as a voluntary standard for food safety to fulfil trade and government regulatory requirements. The National Bureau of Agricultural Commodity and Food Standards is the accreditation body, while the DLD provides implementation functions. Farmers submit their application form and relevant documents to their provincial livestock office which carries out the approval and an annual inspection. The standards range from farm infrastructure, animal feed quality, water quality, farm management, animal welfare, the environment, and animal health management including the use of antibiotics under veterinary supervision.

#### Policy implementation

Department of Livestock Development serves as Thailand’s national veterinary authority under which AFVC officers at national level are responsible for accrediting the RWA and RAU certifications. Staff at regional, provincial, and district levels serve as programme implementing bodies and monitor progress in addition to their routine activities such as animal health controls, veterinary public health controls and disease surveillance. Our document review identified at least five major agro-industrial livestock companies (livestock producers and retaliers) engaged in the RWA programmes, possibly driven by their existing certification as the NSF RWA producers for exporting to EU member states. In 2022, DLD official data reported 214 RWA farms (112 pig and 102 broiler), and 230 RAU farms (83 pig and 147 broiler). The implementation processes for the VOAC programme involve several steps, including setting up an annual plan, application submission, inspection, certification, sample testing, and monitoring.

To establish the annual plan, the AFVC collaborates with regional, and PLO, to probe willingness of pig and poultry farms in their areas to voluntarily participate in the certification. The data collected by the PLO is compiled by the AFVC and included in the annual plan.

Livestock producers who hold GAP certification and wish to participat in either the RWA or RAU certifications can submit an application with the required documents to their PLO. The PLO reviews the application forms and relevant documents and conducts farm inspections to assess if the farms meet the certification criteria. Certificates are granted by the DLD to RWA and RAU farms. In the case of RAU farms, the provincial livestock offices monitor antibiotic use, antibiotic residue, animal health, and management practises on an annual basis.

The DLD applies the existing GAP certification system to facilitate the implementation of the RWA and RAU certifications. Since GAP farms already have designated veterinarians, these veterinarians are also involved in monitoring of RWA and RAU implementation related to the use of antibiotics. DLD auditors responsible for GAP certification, assigned to regional, provincial and district livestock offices, act as inspectors for RAW/RAU certification. Several areas are assessed, including health management and disease control, housing management, feed tracing to its source, water quality, and medicine usage.

In the course of inspections, samples are gathered for testing. Specifically, for the RWA programme, examinations include testing samples of animal feed and water for antibiotic presence. Additionally, meat product samples from both slaughterhouses and retailers undergo annual testing to detect antibiotic residues as part of the RWA certification. For the RAU certification, the farm veterinarian records antibiotic use and the auditor compares the use of antibiotics in the current cycle with the previous one using the mg/Population Correction Unit measurement during the annual inspection.

Samples collected are sent to regional livestock laboratory centres or laboratories owned by the Bureau of Quality Control of Livestock Products. The regional livestock office compiles the laboratory results and submits them to the AFVC. Based on the results, the AFVC issues one-year RWA certificates (renewable annually upon compliance to the requirement) or a three-year cerfication for RAU farms. The regional and provincial livestock offices are responsible for the overall field implementation.

*‘… I (a provincial livestock officer, author) provided information of the programme to farmers. When I received a number of farms that are willing to join, I submitted it to the regional livestock office, and then to AFVC…’* [A11, male, age 50 years].

*‘…AFVC discussed with regional and provincial livestock offices to review the previous plan and implementation, as well as a number of GAP certified farms in the region. Then, provincial livestock offices asked farms’ willing to participation in their catchment area…’* [A07, male, age 47 years].

*‘… After compilation of data of participating farms, AFVC sent a number of farms to the provincial livestock office and then the provincial livestock office to implement in accordance with the rules and regulations of the project…’* [A04, male, age 57 years].

In some areas, there was a meeting among AFVC (central level), regional, provincial and district livestock offices to review the implementation progress and challenges.

The DLD will also grant RWA logo to farmers to advertise their RWA products and certificate for farmers.

*‘…RWA and RAU are policies that everyone would like to participate… participating the programmes has no additional cost to farmers. Government supported everything including the laboratory testing and provided them the certificate. It helped the farmers increase the products’ price and market…’* [A10, male, age 36 years]

The implementation costs of the RWA and RAU certifications were covered by the DLD regular budget and no additional staff was recruited for this purpose. These certifications were integrated into the existing GAP certification system.

The RWA and RAU certifications were established to support the GAP-AMR and NSP-AMR implementation, especially to achieve the target set out by Goal 3, to reduce antimicrobial consumption in animals by 30% by 2021. However, a key informant from a PLO reported that he had never heard about the GAP-AMR or the NSP-AMR. On the other hand, a key informant from another PLO correctly described the target of the NSP-AMR of 30% reduction in antimicrobial consumption in animals. None of the key informants could provide specific details on how the monitoring or evaluation of the RWA and RAU certifications would be conducted.

‘… *I’ve never heard about the plan on AMR before. Is it the national plan or policy of the Ministry of Agriculture and Cooperative or DLD?…*’ [A04, male, age 57 years].

‘…*We have no data on how the farms contribute to the reduction on antibiotic use in the country. It must be the central authority’s work*’ [A09, male, age 45 years].

### Policy content

The policy content is aligned with the goals of the NSP-AMR, by aiming to control, prevent, reduce or slow down the emergence of AMR in animals. The RWA and RAU certifications intend to contribute to this objective by reduce AMU in food production. Indeed, the RWA certification strictly prohibits the use of antibiotics at any stage of the production cycle, while the RAU certification allows for the use of antibiotics, but certified farmers need to demonstrate a reduction in antimicrobial use. Table 3 summarises the objectives and requirements of the two certifications.

## Discussion

The policy analysis of voluntary RWA and RAU programmes in swine and poultry production yields several insights for national and international audiences. The adoption of VOAC involves collaborations between the DLD and the private sector. The programmes are implemented by national, regional, and provincial livestock offices, but the monitoring and evaluation of the programme to address challenges remains unclear.

The commitments of provincial livestock officers to encouraging farmer participation and to annual monitoring of farms and slaughterhouses were identified as a key success factors. The adoption of VOAC was shaped by requirements by countries which are food importer such as European Union; consumers demand quality and safe food as is the case of EU regulation. In 2022, official data from the DLD indicated the presence of 214 RWA farms, consisting of 112 pig farms and 102 broiler farms. Additionally, there were 230 RAU farms, comprising 83 pig farms and 147 broiler farms. Statistics from DLD does not provide geographical location, the annual production capacity of each broiler or swine farm participated in RWA and RAU.

The challenges with labels featuring animal raising claims like RWA, such as consumer confusion, lack of universally accepted definitions, and credibility issues with voluntary, process-based label claims in general, are underscored by instances such as the removal of Tyson’s label claims by the U.S. Department of Agriculture (35). This is particularly relevant given the participation of large agro-industrial conglomerates in the RWA and RAU programmes, which adopt a vertically integrated approach encompassing various stages of livestock production. Despite the potential benefits, such as positive corporate image and brand reputation, promotional efforts in Thailand face hurdles due to limited awareness among consumers about the RWA-DLD programme and antibiotic-free products, attributed to inadequate policy communication by DLD. Consequently, only select companies opt to display the NSF-certified RWA logo on their products.

The publicly available data on total sales and margins of premium products resulting from these programmes, compared to conventional production methods, is currently unavailable. Increased consumer demand for food safety and willingness to pay for premium prices can serve as an incentive for producers and retailers to participate in the programme. Besides international standards like the NSF, other private food standards are gaining significant attentions in other countries, for example large United Kingdom supermarket chains setting contractual standards for livestock producers and prohibiting routine preventive antibiotic use (36).

A Swiss study reveals that the willingness of dairy and veal calf fattening farms to voluntarily reduce or ban antibiotics is influenced by factors such as extra compensation and additional investments (37). Our study lacks concrete data on economic benefits for farmers participating in VOAC, such as cost savings from reduced AMU and potential premium prices for animal products through RWA and RAU. Moreover, some key informants were hesitant to disclose financial information, including cost savings associated with reduced antibiotic use, improvements in feed conversion ratios, or reductions in animal mortality rates in the programmes. While Thailand VOAC and Swiss study share similar objectives of reducing or ban use of antibiotic, our study did not have robust evidence and transparency regarding economic benefits.

The participation criteria for both RWA and RAU programmes include obtaining GAP certification, which requires having a designated farm veterinarian responsible for overseeing antimicrobial use. Consequently, farms lacking GAP certification were deemed ineligible for participation in these programmes. Such stringent requirement rendered the programmes inaccessible for small-scale farms grappling with financial constraints, presenting a notable barrier to their involvement.

Between 2020 and 2022, Thailand faced significant challenges due to the outbreak of Porcine Reproductive and Respiratory Syndrome (PRRS) and African Swine Fever (ASF). In response, certain RWA farms found it necessary to reintroduce antibiotics to manage secondary bacterial infections. This circumstance had a negative impact on the number of pig farms engaged in the RWA certification, contributing to a noticeable decrease. These fluctuations underscore the dynamic challenges from disease outbreaks and potential fragile bio-safety standards among farms.

Our in-depth analysis indicates that the RWA and RAU certifications played, at most, a minimal role in the overall antimicrobial consumption, supported by the limited enrollment of farms, a majority of which were already the NSF RWA certified. However, despite its limited effectiveness, these certifications offer positive aspects of the certification. The RAU certification has potential for scaling up with a higher impact if its challenges are fully addressed and if outcomes such as cost savings from reduced antimicrobial use, increased productivity, changes in mortality rates, and economic gains are thoroughly analysed and disclosed. Given the current deficient statistics from DLD regarding the VOAC programme, it is challenging to attribute the RWA and RAU certifications alone to the achievement of the national goal of a 30% reduction in antimicrobial consumption from 2017 to 2021. However, there has been an overall national level reduction in antimicrobial consumption in food-producing animals from 658.7 in 2017 to 421.5 mg/PCU_Thailand_ in 2020 (a 36% reduction) (38). Further, lack of consumer awareness campaign to recognise for DLD RWA logo for poultry and swine products, while widespread presence of the NSF RWA logo in the market, may contribute to the lack of incentives for more farms to participate in the VOAC programme. Furthermore, there is no monitoring of the market size of RWA and RAU products, which could potentially limit further advocacy efforts. Recognising consumer preferences as potential game-changers is essential, with studies indicating a willingness to pay higher prices for antibiotic-free products in the United States (39) and Europe (40). Focusing on scaling up consumer awareness in Thailand is pivotal, especially considering the limited awareness highlighted by the 2021 Health and Welfare Survey, only 22% of Thai households were aware about RWA products (41).

### Limitations of the study

One key limitation of this study revolves around the representativeness of viewpoints and experiences among informants, especially farmers involved in the VOAC, which may affect the validity of the findings. Specifically, our study only included interviews with two farm managers from large scale production industries. Their view may be positively biassed towards the VOAC implementation feasibility, given their access to advance technologies, large capacity and resources, particularly in terms of farm bio-security, hygiene and sanitation which are enabling factors of low level of mortality when antibiotics were not used. Additionally, the limited farm-specific context provided by the two key informants such as animal health management limits the deep insights of programme implementation.

Moreover, the study’s scope is constrained by the exclusive participation of only two animal producers actively engaged in RWA and RAU certifications. Despite efforts to enlist more farmer informants, reluctance to participate was encountered, with reasons for non-participation remaining undisclosed. It is speculated that large-scale farms may withhold their implementation techniques due to competitive market dynamics. Additionally, the absence of small-scale farm participation in the VOAC, primarily due to their lack of GAP certification, further contributes to the limitation.

The limitation of this study is compounded by the absence of publicly available statistics detailing the total number of RWA and RAU farms, encompassing both broilers and swine, along with their annual production capacity in different years. This lack of data further complicates our efforts to assess the broader impact and discern any trends in the coverage of VOAC. Additionally, the dearth of specific information regarding the locations of these farms presents significant challenges in comprehensively evaluating the distribution and impact of these programmes. Furthermore, external factors such as disease outbreaks in poultry and swine also influence the implementation of VOAC, contributing to the complexity of our analysis. Consequently, this limited insight impedes our understanding and hinders strategic planning, comprehensive evaluation, and programme improvement initiatives.

Further research is imperative to evaluate various aspects related to reduced antibiotic use and the potential economic implications associated with RWA and RAU certifications in Thailand. Specifically, there is a need to investigate the investment costs, as well as the economic benefits and incentives associated with the adoption of these certifications. Additionally, exploraing the premium prices of RWA and RAU products in the market would provide valuable insights into consumer preference and market dynamics. Conducting such researches will contribute to a better understanding of the overall impact and sustainability of these certification programmes in the context of Thailand’s agricultural sector.

### Recommendations

Our study is constrained by the challenge of determining the extent of farm participation, compounded by a dearth of hard, comprehensive and publicly available statistics. This challenge impedes our ability to fully comprehend the impacts of the RWA and RAU programmes on overall antimicrobial consumption and the overall effectiveness of these initiatives. Given these limitations, this study offers several recommendations

#### Optimising the certification strategy

In optimising certification strategies, it is recommended to maintain RWA and RAU certification if there is demonstrated farmer interest in DLD certification over the NSF, take into account potential investment costs and economic gains by farmers. Further investigation into farmer motivations and barriers for RWA and RAU certifications enrollment is crucial. Future studies need to address data gaps, particularly the economic impacts on farmers including mortality and productivity, quantifying actual antimicrobial usage reduction and implementing more effective promotion strategies, will provide evidence for policy decisions and assist farmers in RWU and RAU.

#### Enhancing monitoring and evaluation

The DLD should strengthen its monitoring and evaluation mechanisms for the VOAC programme to address existing challenges effectively. This includes regular monitoring of farm participation and annual assessments of programme outcomes by provincial livestock officers.

#### Increasing communication and consumer awareness campaign

The DLD should prioritise improving policy communication efforts to increase awareness of the RWA-DLD certification and antibiotic-free products among Thai consumers. This can contribute to fostering consumer trust and demand for such products in the market. DLD should launch a consumer awareness campaign to promote the RWA logo for poultry and swine products, thereby incentivising more farms to participate in the VOAC programme.

## Data availability statement

The original contributions presented in the study are included in the article/Supplementary material, further inquiries can be directed to the corresponding author.

## Ethics statement

The study was approved by the Institute for the Development of Human Research Protections for research ethics clearance (Ref.no. IHRP2021168). The interviews were conducted in Thai through video conference and audio-recorded after verbal consent.

## Author contributions

AL: Conceptualization, Formal analysis, Funding acquisition, Investigation, Methodology, Validation, Visualization, Writing – original draft, Writing – review & editing. SKi: Formal analysis, Investigation, Methodology, Validation, Writing – original draft, Writing – review & editing. WK: Formal analysis, Investigation, Methodology, Validation, Writing – original draft, Writing – review & editing. SKh: Formal analysis, Investigation, Methodology, Writing – original draft, Writing – review & editing. RM: Conceptualization, Supervision, Validation, Writing – original draft, Writing – review & editing. VT: Conceptualization, Formal analysis, Investigation, Methodology, Supervision, Visualization, Writing – original draft, Writing – review & editing.
