# Supplementary material for: Voluntary optimisation of antimicrobial consumption in swine and poultry production in Thailand: a policy analysis
Source: Front Vet Sci. 2024 Jul 10;11:1375127. doi: 10.3389/fvets.2024.1375127 (PMC11267447; doi:10.3389/fvets.2024.1375127)
Supplement: Supplementary file 1 [file Data_Sheet_1.docx]

# Supplementary document 1

**Table A1** Policy analysis framework and key questions to be addressed on RWA and RAU in swine and poultry production

| Questions to be addressed | |
| --- | --- |
| A. Policy content |  |
| - Goals and objectives of the policy | - What are the VOAC goals and objectives? - What are the strategic plan and actions which support policy implementation? - When was this policy formulated? |
| - Nature of the policy | - How does the policy affect stakeholders (global, country, ministry, province, organization and farms), any changes? - Are there laws, regulations, supportive measures, incentives to facilitate successful implementment of RWA and RAU certifications? |
| - Clarity of policy | - Is the policy statement clearly defined, notably objectives, goals, implementation plan and M&E systems? |
| B. Policy context |  |
| Situational/ Structural/ Cultural/ Environmental factors | - What external and intenal context when the VOAC was transcended, modified and translated into RWA and RAU Certifications? - How did economic considerations from AMR influence the decision on VOAC programme? - How do international agencies influence the national VOAC programme? |
| C. Policy process |  |
| 1) Agenda setting:  Problem stream ,  Political stream,  Policy stream | - In the last five years or so, what are AMR's events or report of excessive use of antimicrobials in food producing animals, how many of the AMR events transmitted from animals to humans which demanded policy actions?  - Did these problems attract public or media attention?  - How and when did VOAC come to policy attention?  - What is the stakeholders’ viewpoints on the needs for multisectoral collaboration approach to AMR?  - What are the senses of urgency by stakeholders in addressing AMR and why VOAC became one of the potential solutions?  - Is there any evidence demonstrates public supports to VOAC?  - Did political actors support VOAC as a potential solution?  - Is there any documents support the VOAC concept? |
| 2) Policy formulation | - Which actors were involved in formulating RWA and RAU? - What are the stakes which gradually shape VOAC and concept into practical RWA and RAU certifications? - Was RWA and RAU bottom up generated by food animal producers, or top down by DLD? - What are nature of actors’ relationship between government organizations, private sector and government? - Was there influences from outside agencies such as WOAH and FAO, and if yes, through what channels? |
| 3) Policy implementation | - How RWA and RAU certifications were implemented, by whom and through what mechanisms? - Was there budget allocation to support RWA/RAU implementation? - What factors contribute to successes or failures of implementation? |
| D. Policy outputs and outcomes | What are the activities conducted by implementers to facilitate implementation of RWA and RAU? |
| - Antibiotic consumption | Volume of antibiotic consumption in pigs and poultry in the RWA/RAU certifications? |
| - Animal health outcomes | What are the mortality of pigs and poultry in the RWA and RAU certifications?  What are the feed conversation rate in RWA and RAU certifications? |
| - AMR | Prevalence of AMR in pigs and poultry in the RWA and RAU certifications. |

**INTERVIEW GUIDE**

**Topic**: Voluntary optimisation of antimicrobial consumption (VOAC) in swine and poultry production in Thailand: a policy analysis

**Research team**:

Angkana Lekagul

Supapat Kirivan

Wimonrat Tanomsridachchai

Wanwisa Kaewkhankhaeng

Saowapa Khotchalai

**Objectives of the study**

1. To review documents on the RWA and RAU certifications in swine and poultry production
2. To analyse content, context, process, and actors of RWA and RAU certifications in swine and poultry production
3. To explore implementation, output and outcome of the two policies in swine and poultry production
4. To identify gap and recommend policy options to strengthen implementation of the two policies in swine and poultry production

# SECTION 1. GENERAL INFORMATION

**Name of key informant**………….……………………………………………………………

**Position**…………………………………………………………………………………………

**Name of interviewer**……….………….………………….…….………………….…….….…

**Category of key informant**

- Group A: Policy maker or officers responsible for animal health
- Group B: Farmer or animal producers
- Group C: Animal product traders or retailers
- Group D: Farm veterinarians

# SECTION 2. CONTENT AND CONTEXT OF VOLUNTARY OPTIMISATION OF ANTIMICROBIAL CONSUMPTION IN SWINE AND POULTRY PRODUCTION IN THAILAND

## **2.1 Content**

Quality of policy

1. Have you heard about “voluntary optimisation of antimicrobial consumption/VOAC”?

<If the key informant states ’yes’ continue asking item 2) and 3). If no, provide the information about VOAC and move to 3.2 >

1. What is the meaning of “VOAC” in your perspective/interpretation?
2. Have you heard about “voluntary optimisation of antimicrobial consumption/VOAC”?

Can you please provide information on

- 1. Objective
  2. Goal
  3. Plan

1. Do you think the VOAC was clearly defined in the following terms?

## **2.2 Context**

2.2.1 Situational and cultural factors

- Do you know the national contexts which migh influence VOAC adoption?

2.2.2 Economic factor

- In your opinion, how did economics problems caused by AMR influence the emergence of VOAC?

2.2.3 Environmental factor

- In your opinion, how do the international agencies influence the VOAC?

# SECTION 3. PROCESS OF VOLUNTARY OPTIMISATION OF ANTIMICROBIAL CONSUMPTION IN SWINE AND POULTRY PRODUCTION IN THAILAND

## **3.1 Agenda setting**

(Focusing on the problem stream: stakeholders’ interest in AMR problem and value of the problem streams)

1. In the last five years or so, do you know any AMR events or reports of excess use of antimicrobials in animals
2. How many of AMR transmitted from animals to human which may need VOAC policy to address them?
3. Do you think AMR problems attract public and media attention and how problems attract public and media attention?
4. What do you think about the importance of multisectoral collaboration to solve AMR?

## **3.2 Policy formulation**

3.2.1 Actors

1. Do you know any stakeholders or organizations involved in the formulation of VOAC?
2. In your opinion, what are the benefits and risks to certain stakeholders or organizations, which gradually shapes the needs for and the nature of VOAC?

3.2.2 Mechanisms

1. Do you know when and how the VOAC concept emerged and has evolved in Thailand?
2. In your opinion, was policy formulation generated from local needs or were there any influences from outside agencies? Or the combination of both national and international influences? If yes, through what means or approaches?
3. Do you know why VOAC was translated into RWA and RAU

## **3.3 Policy implementation**

3.3.1 Institution and relationship across stakeholders

Institutional relationship

1. In your opinion, how are the relationships (such as collaborative, supportive, neutral, oppose, competitive collaborative) between the following:
   1. Governmental organizations
   2. Private sector and governmental organizations
   3. Local and national levels?
2. From your experience, how can collaboration between organizations be improved?

If the key informant being interviewed belongs to Group B in **SECTION 1**, SKIP TO **SECTION 5**. If not, CONTINUE THE INTERVIEW WITH THE QUESTIONS BELOW.

3.3.2 Implementation approach

Top-down approach vs bottom-up approach

1. How did the VOAC transcend from national to local implementation level?
2. How did the national level communicate VOAC support the local level implementatio
3. From your experience, was there any miscommunication (a failure to get a message across or lack of clear communication) of the VOAC?

International influence

1. What are the roles of international agencies in VOAC implementation in Thailand?

Global policy transfer

1. How did the VOAC policy transcend from international level to country level?

3.3.3 Resources for implementation

3.3.3.1 Technical resources

Human capacity

1. Do you think your current workforces have difficulties or increased workload to accommodate an additional VOAC?
2. Has your organization got new positions for VOAC work?
3. From your perspective, what skills and competency should the officers have to support VOAC implementation? Does your organization have enough skilled personnel for this purpose?

Infrastructure

1. From your perspective and experience, does the existing infrastructure (equipment, lab, etc.) support VOAC implementation?

Capacity development

1. Do you or your organization conduct any training related to VOAC and its implementation?

3.3.3.2 Managerial resources

Budget allocation

1. Did the organization responsible for VOAC allocate additional budget for implementation to local offices? If yes, did the budget adequate? If no, what budget was mobilized to support VOAC implementation?

Personnel appointment

1. Do you or your organization appoint (new) personnel for VOAC implementation?

Structure of process

1. How does the structure of your organization support VOAC process of implementation?
2. Is the structure of your organization clearly defined?

# SECTION 4. GAPS AND RECOMMENDATIONS

1. In your perspective, how can the VOAC programme be improved?

# SECTION 5. SUMMARY OF THE INTERVIEW

**Comments: FOR INTERVIEWER ONLY,** use this space to summarize how the interview went, including the mood or feelings of the key informant during the interview session.
